# Alberta Diabetes and Physical Activity Trial (ADAPT): A randomized theory-based efficacy trial for adults with type 2 diabetes - rationale, design, recruitment, evaluation, and dissemination

**DOI:** 10.1186/1745-6215-11-4

**Published:** 2010-01-12

**Authors:** Ronald C Plotnikoff, Kerry S Courneya, Ronald J Sigal, Jeffrey A Johnson, Nicholas Birkett, David Lau, Kim Raine, Steven T Johnson, Nandini Karunamuni

**Affiliations:** 1 School of Education, University of Newcastle, Callaghan, NSW Australia; 2 School of Public Health, University of Alberta, Edmonton, Alberta, Canada; 3 Faculty of Physical Education and Recreation, University of Alberta, Edmonton, Alberta, Canada; 4 Faculties of Medicine and Kinesiology, University of Calgary, Calgary, Alberta, Canada; 5 Faculty of Medicine, University of Ottawa, Ottawa, Ontario, Canada

## Abstract

**Background:**

The primary aim of this study was to compare the efficacy of three physical activity (PA) behavioural intervention strategies in a sample of adults with type 2 diabetes.

**Method/Design:**

Participants (N = 287) were randomly assigned to one of three groups consisting of the following intervention strategies: (1) standard printed PA educational materials provided by the Canadian Diabetes Association [i.e., Group 1/control group)]; (2) standard printed PA educational materials as in Group 1, pedometers, a log book and printed PA information matched to individuals' PA stage of readiness provided every 3 months (i.e., Group 2); and (3) PA telephone counseling protocol matched to PA stage of readiness and tailored to personal characteristics, in addition to the materials provided in Groups 1 and 2 (i.e., Group 3). PA behaviour measured by the Godin Leisure Time Exercise Questionnaire and related social-cognitive measures were assessed at baseline, 3, 6, 9, 12 and 18-months (i.e., 6-month follow-up). Clinical (biomarkers) and health-related quality of life assessments were conducted at baseline, 12-months, and 18-months. Linear Mixed Model (LMM) analyses will be used to examine time-dependent changes from baseline across study time points for Groups 2 and 3 relative to Group 1.

**Discussion:**

ADAPT will determine whether tailored but low-cost interventions can lead to sustainable increases in PA behaviours. The results may have implications for practitioners in designing and implementing theory-based physical activity promotion programs for this population.

**Clinical Trials Registration:**

ClinicalTrials.gov identifier: NCT00221234

## Background

Type 2 diabetes mellitus (T2DM) is a serious chronic disease. The World Health Organization estimates 180 million people worldwide live with this condition. This number is likely to more than double in the next 25 years [1]. The long-term complications of diabetes, such as microvascular and macrovascular diseases, can be delayed or prevented with appropriate interventions, including drug treatment, physical activity (PA), nutrition therapy, and body weight management [2,3].

Physical activity plays a key role in T2DM management [2,4-7]. In addition to providing measurable psychological benefits, physical activity is associated with improvements in cardiovascular risk profile such as increased insulin sensitivity and decreased insulin resistance, reduced body fat, and decreased blood pressure [8]. Participation in regular, moderate intensity physical activity has been reported to decrease glycosylated haemoglobin (A1C) to a level that is associated with a decrease in the risk of diabetic complications [5]. Moreover, higher cardiorespiratory fitness among this population is associated with reductions in morbidity and mortality [9].

Physical activity guidelines designed to improve and maintain health have been developed for this population [2]. These guidelines recommend that people with T2DM participate in moderate intensity physical activity, such as brisk walking and biking for at least 150 minutes each week, over at least three non-consecutive days. It is also recommended that some form of resistance training be incorporated at least three times per week [2]. Current evidence, however, indicates the majority of adults with T2DM remain either sedentary or insufficiently active to achieve health benefits [10,11].

Given the beneficial effects of physical activity for those with T2DM and the low physical activity participation levels among this population, effective strategies to prevent and treat obesity and to encourage physical activity are needed [2]. In this regard, great potential exists in the employment of social-cognitive theories to explain behaviour change and design interventions for diabetic populations [11-14]. More recently, a number of studies among the T2DM population have provided sufficient evidence in defining key social-cognitive determinants of physical activity behaviours among the T2DM population [15-19]. This information provides a valuable platform upon which population-based interventions can be developed and tested.

The mode and efficiency of program delivery are important considerations for physical activity promotion at individual and population levels. For example, tailored messages appear to be more efficacious than generic materials for producing change across various health behaviours as well as the promotion of physical activity [20-22]. The use of printed materials and telephone-based counseling are two methods of applying individually tailored physical activity interventions that have achieved increases in physical activity in the adult population [23-26].

We designed the Alberta Diabetes and Physical Activity Trial (ADAPT) to explore the efficacy of two enhanced behavioural intervention strategies involving different modes of delivery. Using social-cognitive theories, the enhanced interventions were tailored to individuals' stage of readiness and personal characteristics, and were delivered via print-based or telephone-based modes.

The primary aim of ADAPT is to explore the efficacy of three behavioural intervention strategies namely: 1) standard printed physical activity educational materials provided by the Canadian Diabetes Association [i.e., Group 1/control group)]; (2) the same standard PA educational materials provided to Group 1, pedometers, log book and printed PA information matched to individuals' PA stage of readiness [27] provided every 3 months (i.e., Group 2): and, (3) PA telephone counseling protocol matched to PA stage of readiness and tailored to personal characteristics, in addition to the materials provided in Groups 1 and 2 (i.e., Group 3).

### Specific Objectives

The objective of this study was to compare the efficacy of two enhanced behavioural intervention strategies for the promotion of physical activity versus a standard materials/control group in the adult general T2DM population.

## Methods/Design

### Selection Criteria (eligibility)

Men and women aged 18 years and older, diagnosed with T2DM, with regular access to a telephone, and without an English language barrier were eligible to participate. Participants were initially screened at baseline using the Canadian Society for Exercise Physiology's Physical Activity Readiness Questionnaire (PAR-Q; http://www.csep.ca/english/view.asp?x=698); anyone who answered 'yes' to any question were asked to visit their doctor to ensure they were safe to start a physical activity program.

### Primary outcomes

*Physical activity behaviour *was assessed by subjective and objective methods. Self-reported physical activity was collected using a slightly modified version [10] of the validated Godin Leisure-Time Exercise Questionnaire [28] that asked participants to report the average number of times per week and average duration, in the past month, they engaged in vigorous (rapid heart beats, sweating), moderate (not exhausting, light perspiration) and mild (minimal effort and no perspiration) intensity physical activity for a minimum of 10 minutes per session. Participation responses for the vigorous and moderate activity categories were then added to obtain summary scores of weekly PA and include weighted and non-weighted scores for PA intensity (i.e., weekly moderate intensity minutes multiplied by 4 Metabolic equivalents and vigorous intensity multiplied by 7.5 Metabolic equivalents) [29]. Mild intensity activity responses were not included in the calculation since this level of intensity has no proven association with health benefits and is not included in the current practice guidelines.

As an objective measure of PA, total daily steps were collected with a pedometer over 3 days. Participants were instructed to wear a pedometer during waking hours and to record the total daily step counts in a log.

The study's second primary outcome, glycosylated hemoglobin (A1C), was measured using a turbidimetric immunoinhibition method on a Synchron LX20 analyser (Beckman Coulter, Fullerton CA) [30].

### Demographic and Secondary outcomes

*Socio-demographic factors *were measured using questions based on the Statistics Canada 2001 census survey [31] and included: age, sex, ethnic origin, marital status, educational level, and gross annual family income.

*Health factors *were assessed using previously published self-report measures [10,32] to determine diabetes type, height and weight (used to calculate body mass index); daily use of insulin or oral antihyperglycemic medication; age of diagnosis, cardiovascular disease (angina, past myocardial infarction) and cardiovascular disease risk (elevated blood pressure, cholesterol levels).

*Clinical variables *(biomarkers) were measured after an overnight fast; insulin, glucose, total cholesterol, high-density lipoprotein-cholesterol (HDL), and triglycerides were measured using enzymatic methods, and low-density lipoprotein cholesterol (LDL) was calculated using the Friedewald equation without use of the preparative ultracentrifuge. More specifically, insulin was measured using a Roche Diagnostics Elecsys 2010 system using the sandwich principle [30]. Glucose was measured using a hexokinase technique [30]. Plasma lipids, glucose and A1C were determined on a Synchron LX20 analyser (Beckman Coulter, Fullerton CA). Additionally, plasma C-reactive protein was analyzed using a turbidimetric technique and an automated Nephelometer Analyser System (Behring Diagnostics, Mannheim, Germany) using anti-CRP mouse monoclonal antibodies with latex microparticles.

*Social-cognitive variables *were collected by questionnaire and Table 1 provides a summary of the social-cognitive theories and related model constructs with the already calculated baseline reliability estimates (Cronbach α).

**Table 1:** ADAPT psychosocial questionnaire reliability summary (N = 287)

Construct (theory)	Number of items	Cronbach's α at baseline	References
Planning/Implementation (SCT)	7	.88	[16]
Intention (SCT)	4	.79	[16]
Outcome expectations (SCT)	8	.90	[16]
Pros (SOC)	5	.81	[35]
Cons (SOC)	3	.72	[35]
Attitude (TPB)	6	.86	[15]
Subjective Norm (TPB)	4	.73	[15]
Perceived behavioural control (TPB)	2	.56*	[15]
Response Efficacy (PMT)	3	.84	[18]
Severity (PMT)	1	NA	[18]
Vulnerability (PMT)	1	NA	[18]
Self-Efficacy (TPB/PMT/SCT/SOC)	12	.94	[16,18,35]

**Note**: PMT = protection motivation theory; TPB = theory of planned behaviour; SCT = social cognitive theory; SOC = stages of change

*Pearson correlation

*Health related quality of life (HRQL) *was assessed using the SF-12 Physical and Mental scales [33] and the EQ-5D scale [34].

### Design

**Figure 1:**
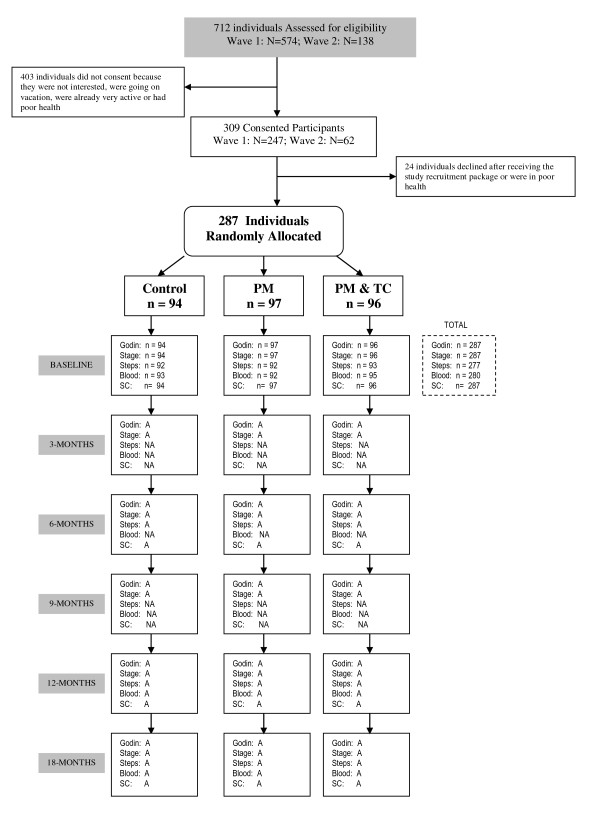
**Study flow diagram for ADAPT**. PM = print material; TC = telephone counseling; SC = social-cognitive variables; A = to be assessed; NA = not to be assessed.

287 participants were randomly assigned to one of three intervention groups. Group 1 was the standard material/control (i.e., current Canadian Diabetes Association clinical practice physical activity information and was not stage-matched). Group 2 received the same standard material as Group 1 and print media that was stage-matched (i.e., Precontemplation, Contemplation, Preparation, Action, or Maintenance) [27] to baseline, 3, 6 and 9 month assessment and appropriate to the season (e.g., Spring, Winter), a pedometer and an activity log. Group 3 received all materials as in the other study arms, and additionally received physical activity specific telephone counseling. All groups received the print materials via regular mail. Figure 1 provides a visual flow diagram of the study including data collection time points. The 'Theory Guidance' section below explains the theoretical underpinnings of the stage-matching.

### Intervention Strategies

All groups received a research package before randomization, which included an information letter detailing the study design and objectives, a consent form and the Canadian Society for Exercise Physiology's Physical Activity Readiness Questionnaire (PAR-Q) to assess the need for physician permission to participate in the study http://www.csep.ca/english/view.asp?x=698. At the time of recruitment, it was clearly articulated to participants that ADAPT was a physical activity study and that they would be randomized (see below) into any one of the above-defined groups.

Once consent was received from the participants, they were randomized into one of the study's three arms (see the 'Recruitment and Randomization' section below for details on the randomization procedure). In order to record average steps for comparison, all participants in all three intervention groups were sent a pedometer. All participants were instructed to record pedometer steps for 3 days at the baseline, 6, 12 and 18 month assessments. The standard/control group was instructed not to use the pedometers between the assessment periods. All groups received a tape measure with instructions (which included an instruction photo) to self-measure waist circumference and report height and weight used to calculate Body Mass Index (BMI). The following details the individual interventions for each of the groups:

### Group 1

Group 1 was classified as the standard care/control group (n = 94). This group received a one page leaflet outlining the Canadian Diabetes Associations' physical activity guidelines.

### Group 2

Group 2 was the print material group (n = 97). This group also received the Canadian Diabetes Associations' physical activity guidelines as well as stage-based print materials developed to address issues specific to the stage in which they were currently assessed. These materials were also tailored to be season-specific (i.e., Winter, Spring, Summer and Fall versions) and were mailed every 3 months. The purpose of the materials was to help address some of the specific issues related to each stage (i.e., Precontemplation, Contemplation, Preparation, Action and Maintenance). Therefore, based on the 5 stages and 4 seasons, 20 combinations of booklets were available for supporting stage progress or retention. Participants in this group also received a pedometer and tape measure. Finally, participants were given a fridge magnet calendar and a dry erase marker in order to chart progress.

### Group 3

This intervention group (n = 96) received the same intervention materials as specified in Groups 1 and 2. In addition, this group also received telephone counseling designed to give additional support to participants by trained counselors. Calls took place over the length of the 12-month intervention. For the first month calls were weekly, the next month calls were bi-weekly, then for the remainder of the intervention calls were monthly. This tapered schedule was to allow participants to become less reliant on counselor support as the intervention progressed.

Training for five telephone counselors (two health promotion graduate students and two psychology graduates) took place over a one day 7 hour training course. Training was completed with two project coordinators. The counselors were trained in seven key areas: the helping process, methods of communication, areas of influence, psychological underpinnings for behaviour change, the Transtheoretical Model (TTM) [27], an overview of T2DM, physical activity and older adults, as well as physical activity related to T2DM and motivational interviewing techniques. Telephone counselors were also given a presentation and materials from the Canadian Diabetes Association. Counselors also received the Canadian Diabetes Association 2003 Guidelines for Physical Activity, Canada's Physical Activity Guide to Healthy Living, summarized research literature on PA and diabetes, pamphlets provided by the Canadian Diabetes Association giving a general overview on T2DM: medication, PA guidelines, diet, foot care, blood glucose testing, diabetic complications, and important lifestyle changes. All materials developed for this trial are available from the Principal Investigator (RCP) and are located on the Physical Activity and Population Health Research Laboratory web site: http://www.uofaweb.ualberta.ca/chps/paph.cfm.

### Theory Guidance

To enhance physical activity stage-matched interventions, Plotnikoff and colleagues examined social-cognitive constructs and items from the Theory of Planned Behaviour, the Health Belief Model, Protection Motivation Theory, Social Cognitive Theory, and the Transtheoretical Model in a large longitudinal, randomized population-based adult sample, to predict forward physical activity behaviour stage of change transition [35-38]. From these results, there now exist strong data validating the magnitude of theoretical robustness for a number of social-cognitive constructs to predict (or not predict) stage transition. For example, Courneya and colleagues have demonstrated certain Theory of Planned Behaviour constructs outperform constructs from the Transtheoretical Model spanning a number of stage transitions [35,36]. Taken together, this work served to guide the development of an *integrated Stage-based Model *(ISM) which guided the development and testing of stage-matched materials [39]. The study's stage-matched materials (booklets) were then modified and pre-tested with adults with type 2 diabetes prior to being employed in ADAPT.

### Recruitment and Randomization

A multi-strategy approach was taken between October 2005 and January 2006. Recruitment was targeted to those willing to participate. General advertising strategies were used across the province of Alberta, Canada. Advertisement posters were placed within the local health region sites (i.e., hospitals and community clinics), at regional Diabetes Education Centres in two main urban centres, and at community pharmacies. Advertisements were also placed in the Canadian Diabetes Association national newsletter and on the website. The principal investigator (RCP) completed an interview that was repeatedly broadcasted on a local cable television station. Further, advertisements were placed in newspapers across urban and rural communities. Finally, participants of a previously completed non-intervention survey who consented to be approached to participate in future physical activity studies were contacted by mail, telephone and email [10]. Ethical approval was granted from an institutional review board at the University of Alberta, Edmonton, Canada.

We employed blocked randomization with a block size of '3'. A Fortran program (subroutine) was used to generate random permutations of the numerals 1-2-3. These generated numbers were used to assign the participants into the three study groups. That is, after every third subject, we were assured that equal numbers had been assigned to each of the treatment arms (since the subroutine generated permutations of 1/2/3). The person recruiting to the trial was unaware of the block size of 3. Stratification was not used in the randomization process, as we did not anticipate any specific variables to have a major influence on the outcome.

Allocation of study participants was concealed to all study investigators. Only one Research Assistant, who was not directly involved in the study, had access to the database and the coding of the three study groups. After all the recruited individuals were randomized by the above-mentioned Research Assistant, the participant's group assignments along with their study IDs were passed on to the research coordinator. Research staff (other than the above Research Assistant) and investigators were unaware of the treatment allocation prior to randomization. Participants were notified by telephone of their group allocation. A log was maintained by the Research Assistant of all randomization encounters.

This study used a Relational Database Management System (RDBMS; Microsoft Access) to organize the large quantity of data generated. In addition to information such as group allocation, contact information, drop-out information, date questionnaires were returned, and blood data received, this data management method records multiple items for each individual such as comments of individuals relating to the study, any changes in their medical condition, if new pedometers were sent and when, if log books were returned, etc. (This was done by establishing what is known as "one-to-many" data table relationships in the Access RDBMS.) This system was developed and maintained by the above-mentioned Research Assistant who was not directly involved in the study. Any changes to the database were preserved in writing to show what was changed and the reason for any changes. The generation of multiple (all) versions of the database as changes occur, will be permanently archived as to ensure a complete audit trail.

The Relational Database Management System facilitated generating lists of individuals (in the form of customized database queries and reports) for those who did not return questionnaires, or of individuals from whom blood data has not yet been received, etc. These lists were used to contact individuals and encourage participants to stay in the study. It was also possible to generate mail labels and employ merge (e-mail) to target specific customized groups who did not respond. After each questionnaire mail-out, a reminder call was given to each participant two weeks after the initial mail-out. Three attempts were made to directly talk to the participants; and failing this, a message was left on their answering machine. An email was also sent if an email address was available for the participant. After another two weeks, a questionnaire package was re-sent to any non-respondents. Further, any participants who chose to withdraw were sent a final letter acknowledging their decision to withdraw and a final opportunity to reconsider staying in the study. Other methods to increase study retention included giving participants an option to take part in the Wave 2 of the study (group of study participants who joined the study three months later) if they were unable to commence for the Wave 1 group.

### Analysis Strategy

Physical activity data, clinical data, and HRQL measures will be analyzed using Linear-mixed model (LMM) analyses employing intention-to-treat principles [40] using the Statistical Program for Social Sciences (SPSS) 16. LMM analyses examine changes from baseline to each follow-up time point for each intervention group compared to the changes for the same time period for the control group [41] (See Appendix A).

LLMs use all available data and provide a valid analysis when data are missing at random. Tests will be conducted to examine if missing data are missing at random. Suitable multiple imputation methods will be utilized if the data are found to be not missing at random. A total of three planned comparisons will be completed and include: Group 1 **to **Group 2, Group 1 **to **Group 3, and Group 2 **to **Group 3. The alpha levels will be reported along with effect sizes (following Cohen's guidelines) for each of the analyses [42].

Prior to analyses, any baseline differences between groups for variables (based on the literature) that may be associated with activity levels of participants will be assessed for baseline balance. These include, age, gender, level of education, income, marital/partner status, diabetes duration, BMI, medication (i.e., insulin), physical disability index, and A1C levels. Depending on the variables (categorical or continuous measures) chi-square tests or one-way ANOVAs will be conducted to assess baseline balance on the above-mentioned variables. Given the inconsistency of these variables in their association of PA in this population [10] the primary analyses will only co-vary for baseline physical activity. However, we will also present adjusted results, controlling for any of these variables displaying significant differences (p < .05) between groups at baseline.

We will compare study dropouts with individuals who completed the study on physical activity levels, demographic and medical variables. We also plan to compare drop-out rates between the different study groups.

### Statistical power and sample size

The study's primary analysis is the comparison of physical activity behaviour between the three study groups, from baseline to the 12-month time-point. Assuming a moderate correlation (r = 0.5) between baseline and post-intervention, to detect a mean difference of 0.5 standard deviation between study groups for the main dependent outcome (i.e., physical activity behaviour) at post-intervention (as suggested by Cohen) [43] the required sample size is 76 participants per group (power = 0.80; alpha = 0.01 [44]. Secondary analyses will examine the PA behaviour change between the study groups across the other study time-points (i.e., 6 and 18 months).

The sample size is also adequate to assess differences between the groups on the study's secondary outcomes of the psychosocial measures and nutrition behaviours, [43] and based on published intervention studies to date, it is more than adequate (power >0. 80; alpha = 0.05) to independently detect differences on the anthropometric and biometric outcomes (i.e., body weight, hip-waist ratio, fitness measures, A1C, fasting glucose, fasting lipid profile, plasma insulin levels, and C-reactive protein).

Anticipating a maximum study drop-out rate of 20% at 12-months (study's primary end-point), and 30% at 18-months we enrolled 287 individuals. The dropout rate was based on conservative estimates from other similar type of investigations on individuals with type 2 diabetes [45] and our study's retention strategies.

## Discussion

We have described the design of a pragmatic trial that draws evidence from several social-cognitive theories combined with various modes of program delivery in an effort to examine the efficacy of population-based (i.e., wide reaching and low cost) strategies for physical activity promotion among adults with T2DM.

A novel feature of our design is the "polytheoretical" approach to behaviour change in this population, which is in contrast to most previous studies that often take a 'one-theory-fits-all' approach. Key constructs from various social-cognitive theories served to guide ADAPT [46-51]. Central to our design was the Stages of Change or Transtheoretical Model [27], and intervention groups were stage-matched (5 stages) and matched based on four seasons, consisting of 20 separate stage-based booklets. Beyond this unique stage- and season-matched approach, ADAPT will provide further evidence for the utility of targeting various psychosocial variables relevant when tailoring theory-based PA interventions. Moreover, the inclusion of constructs from continuous behavioural models (i.e., Protection Motivation Theory, Social Cognitive Theory, and Theory of Planned Behaviour) found to predict forward stage transition, will speak to the potential for an enhanced and integrated stage-based model for behaviour change [39].

The relative advantages and disadvantages of the modes of delivery used in ADAPT (i.e., printed material and telephone counseling) were considered in the development of the trial groups. For example, the benefits of using printed materials to promote behaviour change include: the promotion of self-initiated change; low cost; potential to reach large numbers of individuals; decreased staff and participant burden; minimized time barriers as individuals can read the material when they have time; and the material can be used as a reference tool at a later date [52]. Conversely, the disadvantages to using print materials include: difficulty determining dose-response; the material may not be seen as personally relevant nor engaging; lack of social support due to lack of personal contact; and uncertainty that individuals will read the material [52].

The telephone counseling mode of physical activity promotion may be particularly relevant to a diabetic population as telephone-mediated interventions appear to be effective in specialized populations; particularly those that may be highly burdened by comorbidities such as individuals with T2DM [53,54]. Further, the telephone can provide many of the advantages of face-to-face contact with less of the disadvantages; that is, lower cost, requires less staff time, has a greater reach, and availability in most households [55]. The main disadvantages of using telephone mediated interventions is their greater expense compared to printed materials; and it is possible that any added effectiveness may not be relevant once a physical activity program is established [56].

Pedometer and step log use is an additional attractive strategy to increase the efficacy of physical activity interventions for people with T2DM. Not only are pedometers of relatively low-cost, they offer a dual purpose to researchers in that they provide an objective estimate of daily activity pattern (i.e., walking) and also fit within theory-based physical activity interventions that incorporate goal setting and self-monitoring [57-61]. In one of the first research studies to employ pedometers to increase walking in a small sample of individuals with T2DM, a 10,000 steps per day goal was set. Participants exceeded this goal and on average walked 19,000 steps per day and achieved considerable improvements in insulin sensitivity [62]. Since then, other pedometer-based research in this population suggests that both behavioural and clinical outcomes can be favorably improved [63,64] and for many of these reasons we included this simple tool in ADAPT.

A recent review of available literature on the cost-effectiveness of lifestyle interventions for patients with diabetes concluded that these would likely be highly cost-effective, although the available evidence was limited [65]. In particular, the authors found few comparative effectiveness trials, with only short-term follow-up. They recommended that future research should focus on long-term effectiveness and should compare multiple treatment strategies to determine incremental costs and benefits of one over the other. This was one of our objectives in the ADAPT study, and if either of the two enhanced intervention strategies prove to be more effective, we intend to conduct incremental cost-effectiveness analyses relative to the standard intervention strategies.

Finally, the RE-AIM framework which has been applied to evaluate health behaviour change interventions including those that target physical activity [66] and diabetes [45,67-69] in public health practice, will serve to guide the dissemination and effectiveness of ADAPT in terms of its adoption, implementation and maintenance, in public health settings.

In summary, since the global prevalence of T2DM is expected to increase beyond previous estimates, best-practices for promoting a key cornerstone of T2DM management at a population level are urgently needed. In addition to the T2DM population, the results of this study may provide valuable information relating to other physical activity-based chronic disease prevention and management interventions.

## List of Abbreviations

A1C: glycosylated haemoglobin; ADAPT: Alberta diabetes and physical activity trial; BMI: Body mass index; CDA: Canadian Diabetes Association; CRP: C-reactive protein; EQ-5D: European Quality of life - 5 dimensions; HDL: High density lipoprotein; HRQOL: Health related quality of life; ISM: Integrated stage-based model; LDL: Low density lipoprotein; LMM: Linear mixed model; PA: Physical activity; PAR-Q: Physical Activity Readiness Questionnaire; PMT: Protection motivation theory; RE-AIM: Reach, Efficacy, Adoption, Implementation, and Maintenance; SF-12: Short form health survey -12 item; T2DM: Type 2 diabetes mellitus; TPB: Theory of planned behaviour; TTM: Transtheoretical model; SCT: Social cognitive theory; SOC: Stages of change.

## Competing interests

The authors declare that they have no competing interests.

## Authors' contributions

RCP conceived the study. KSC, RJS, JAJ, NB, DL and KR provided input into the study and intervention design. RCP, NK and STJ were responsible for drafting the manuscript. All authors critically evaluated the article for content and approved the final version.

## Appendix A

LMM model statement is: yij = ÿ0 + ÿ1.tij + ÿ2.gi1+ ÿ3.gi2 + ÿ4.(gi1*tij) + ÿ5.(gi2*tij)+bi0 + bi1 * tij + ÿij, i = 1, ..., 287; j = 1, ..., 6, where ÿ's are the fixed effects coefficients, b's are the random effects coefficients, the product terms gij*tij are interaction terms of group-by-time, and ÿij is the error for observation j in group i. The command (in SPSS) used will be as follows [one group factor (GROUP) and one repeated measures factor (TIME), where ACTIVITY (and other outcomes) is the dependent variable and ID is the subject variable].
